# The Effect of Rainfall on Aquatic Nitrogen and Phosphorus in a Semi-Humid Area Catchment, Northern China

**DOI:** 10.3390/ijerph191710962

**Published:** 2022-09-02

**Authors:** Chen-Yang Shou, Ye Tian, Bin Zhou, Xu-Jin Fu, Yun-Ji Zhu, Fu-Jun Yue

**Affiliations:** 1 Institute of Surface-Earth System Science, School of Earth System Science, Tianjin University, Tianjin 300072, China; 2 Tianjin Eco-Environmental Monitoring Center, Tianjin 300191, China; 3 Tianjin Academy of Eco-Environmental Sciences, Tianjin 300191, China; 4 Tianjin Huanke Environmental Consulting Co., Ltd., Tianjin 300191, China; 5 Tianjin Bohai Rim Coastal Earth Critical Zone National Observation and Research Station, Tianjin University, Tianjin 300072, China

**Keywords:** rainfall, nitrogen and phosphorus, Yuqiao Reservoir, water quality, climate change

## Abstract

The impact of rainfall on water quality may be more important in semi-arid regions, where rainfall is concentrated over a couple of months. To explore the impact of rainfall changes on water quality, e.g., nitrogen (TN) and phosphorous (TP), the diversion from Luan River to Tianjin Watershed in the northern semi-humid area was selected as the study area. TN and TP concentrations in rivers and the Yuqiao Reservoir during the three-year high-flow season (2019–2021) were analyzed. The response relationship and influencing factors among the watershed’s biogeochemical process, rainfall, and water quality were clarified. The results showed that rainfall in the high flow season mainly controlled the river flow. The concentration of TN and TP in the inflow rivers is regulated by rainfall/flow, while the concentration of TN and TP in the water diversion river has different variation characteristics in the water diversion period and other periods. The lowest annual concentrations of TN and TP were observed in the normal year, while the highest annual concentration was observed in the wet year, indicating that the hydrological process drove the nutrient transport in the watershed. For the tributaries, the Li River catchment contributed a large amount of N and P to the aquatic environment. For the reservoir, the extreme TN concentrations were the same as the tributaries, while the extremes of TP concentrations decreased from the dry year to wet year, which was in contrast to the tributaries. The spatial variation of TN and TP concentrations in the reservoir showed that the concentration decreased following the flow direction from the river estuary to the reservoir outlet. Considering climate change, with the increase of rainfall in North China in the future, the TN and TP transport fluxes in the watershed may continue to increase, leading to the nitrogen and phosphorus load of the downstream reservoir. To ensure the impact of the increase of potential N and P output fluxes in the watershed on the water quality of the reservoir area, it is necessary to strengthen the effective prevention and control of non-point source pollution in the watershed.

## 1. Introduction

Water eutrophication caused by excessive nutrient input is one of the current most urgent water resource issues [1,2,3]. Nutrients containing N and P play an important role in many factors causing water eutrophication [4,5,6], and human activities are the main source of these nutrients [7]. Point source pollution and non-point source pollution are the main ways for nutrients to enter the aquatic environment. Among them, the impact of non-point source pollution is often greater than that of point source pollution [8]. At the same time, the wide range of sources, the complex migration process, and the randomness of non-point source pollution make its prevention and control more difficult [6,9]. At present, with the promotion of China’s ecological civilization construction, it is particularly important to control pollutant emissions, strengthen pollution control, and improve environmental quality [10]. Therefore, it is of great significance to explore the variation of TN and TP concentrations in the aquatic environment and analyze their influencing factors for the governance of the water environment and the rational planning of human activities in the watershed.

In the watershed, N is mainly transported and transformed between soil water organisms and the sediment–water interface [11,12]. In addition to being directly absorbed by plants [13], N in the soil will also be leached and infiltrated into groundwater or enter surface water with surface runoff, particularly NO_3_^−^-N [14]. The change process of various N elements in the sediment–water interface includes adsorption, sedimentation, biological nitrification, denitrification, assimilation, absorption, ammonization, and anaerobic ammonia oxidation reaction [15]. Moreover, wet and dry deposition in recent years has become an important contributor to the watershed in many parts of the world, particularly nitrogen by wet deposition [16,17]. P in the watershed mainly enters the soil through fertilization [18], and then part of the solution in the soil enters the plant through plant absorption, forming biologically bound organic P. Another part of the phosphate solution in the soil can be transformed between the adsorbed and free state by over-adsorption and desorption [19,20]. In all the processes of migration and transformation of soil P, P can be leached into groundwater or merged into surface water with surface runoff [21]. Part of the P entering the water body can transform with the fixed or adsorbed P in the sediment. When excessive P enters the aquatic system, a bloom of algae and plankton is normally observed, particularly in eutrophic aquatic systems with a reasonable ratio of N/P [22]. In contrast to TN, TP deposition is mainly considered dry deposition in aquatic environments [23].

At present, the existing studies on the factors affecting N and P in the watershed mainly include land use [24], fertilization intensity [25], landform [26], vegetation cover type [27], and rainfall [28]. The change of biochemical conditions in the environment (pH, redox potential, and biological metabolism) can change the existing forms of N and P and affect the concentrations of TN and TP in water [29]. For example, the enhancement of hydrodynamic conditions can accelerate the migration efficiency of TN and TP [30,31]. In addition, as the major contributor, human activities comprising fertilizer usage in farmland and point sources can significantly increase the concentration of TN and TP in soil and aquatic systems [32,33]. Furthermore, the watershed will determine the total amount of fixed N and P of plants [34]. The above factors are directly or indirectly related to rainfall. Therefore, it is of great practical significance to study the relationship between rainfall changes and TN and TP concentration changes in the watershed for the protection of the ecological environment.

Yuqiao Reservoir is an important regulation reservoir of “the Water Diversion Project from the Luan River to Tianjin City” and an important water source in Tianjin. Due to the frequent human activities in the reservoir watershed, water pollution and water quality deterioration are obvious [35], especially in summer and autumn; cyanobacteria blooms occur frequently in the reservoir, seriously affecting the safety of the water supply in Tianjin. Therefore, it is extremely important to explore the source, migration, and transformation of pollutants in the Yuqiao Reservoir and analyze their influencing factors, which should be considered in the future for the prevention and remediation of pollutants [36]. At present, the research on the water quality of the Yuqiao Reservoir has mainly involved the estimation of reservoir water source, nutrient load through models [37,38], land use types [39], endogenous sediments on the water quality of the reservoir [40,41], and diversion structure and phytoplankton on the water quality of the reservoir [5,42]. There is still a lack of research on the mechanism between rainfall change and water quality against the background of climate change.

Based on the analysis of rainfall levels in different years from 2011 to 2021, this paper selects three representative years of rainfall as dry, wet, and normal years. Through the monitoring data of discharge and quality in the high flow season of the Yuqiao Reservoir Watershed for three years, this paper analyzes the variation characteristics of TN and TP in the tributaries and reservoir. The objectives of the present study are to explore the impact of flow on the water quality of the inflow river and to understand the spatial and temporal variation of water quality in the reservoir. Furthermore, the influencing factors of water quality in the watershed are analyzed to provide scientific evidence for the prevention and control of eutrophication in the watershed.

## 2. Method

### 2.1. Study Area

**Figure 1:**
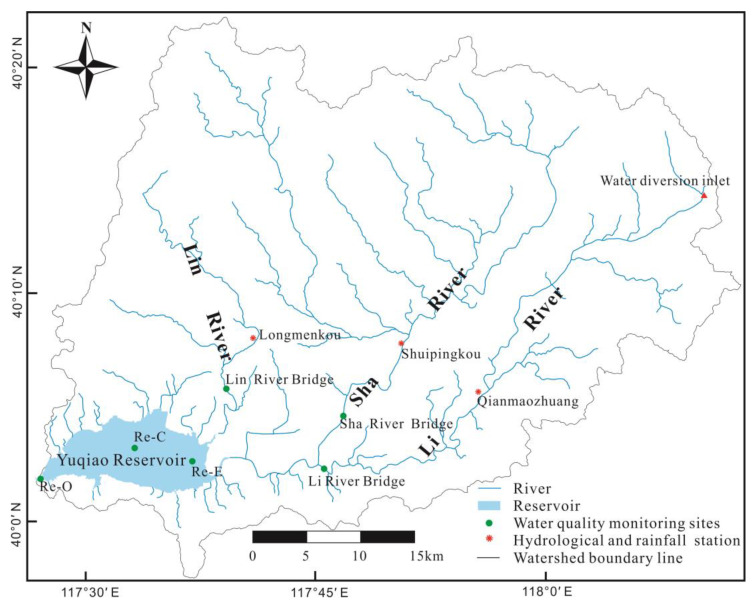
The location of hydrological stations and water monitoring sites in the Yuqiao Reservoir watershed. Re-E, Re-C, and Re-O represent the east, center, and outlet of the reservoir.

The Yuqiao Reservoir (Figure 1) is located in the north of Tianjin (117°27′~117°38′ E, 40°01′~40°05′ N). It is the first large-scale inter-watershed water transfer project in China, with a normal storage area of 86.8 km^2^. The total area of the reservoir watershed is 2060 km^2^, of which 424 km^2^ is in Tianjin [43]. The altitude of the watershed is high in the northeast and low in the southwest. The watershed belongs to a temperate continental monsoon climate with an annual average temperature is 11~12 °C. The main tributaries in the watershed are the Lin River, Sha River, and Li River [38]. Among them, the Li River is the water diversion channel of “the Water Diversion Project from the Luan River to Tianjin City”, which highly regulates the discharge during the water diversion period [44]. The rainfall is mostly concentrated in summer, particularly in July and August [5]. Major water flow is mainly observed in the Lin River, one of the seasonal rivers. Therefore, the discharge of the Sha River can represent the natural response to rainfall change.

### 2.2. Rainfall Characteristics of the Study Area

**Figure 2:**
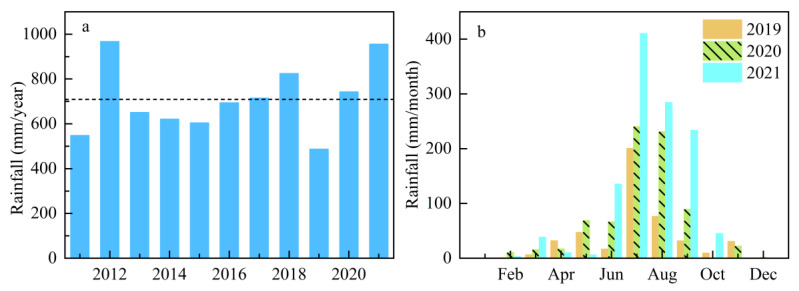
(**a**) The annual rainfall of the study area from 2011 to 2021. Black dashed line represented the mean rainfall of study area; (**b**) monthly rainfall from 2019 to 2021 at Yuqiao Reservoir station.

The annual rainfall of the study area in the recent 11 years (2011–2021) is shown in Figure 2a. The average annual rainfall of the Yuqiao Reservoir Watershed is 709.2 mm. To separate the dry, wet, and normal years, deviation (≥25%) from the mean annual rainfall was used to create them, meaning that a negative deviation can be defined as dry years and a positive deviation is termed as wet years [45]. Therefore, three continuous years—2019 (−31.5%), 2020 (4.7%), and 2021 (34.7%)—were selected for further analysis as the representatives of dry, normal, and wet years, respectively, which may minimize the potential effect from the land use change compared to a large time interval.

According to the analysis in Figure 2b of the monthly rainfall from 2019 to 2021 at the Yuqiao Reservoir station, the rainfall pattern in each year was similar, and the peak value in these three years all occurred in July. The monthly average distribution of rainfall in the year was extremely uneven, and the rainfall was mainly concentrated from June to September, defined as the high flow season, which accounted for 72%, 82%, and 91% of the annual rainfall of the year during 2019 to 2021, respectively. Therefore, the high flow season rainfall in the Yuqiao Reservoir watershed can represent characteristics of the annual rainfall level. The corresponding discussion on the change characteristics of water quality over three years to the impact of rainfall is mainly focused on the high flow season.

### 2.3. Data of TN and TP

To obtain research data, rainfall and flow data were respectively taken from the daily monitoring data of Qianmaozhuang, Shuipingkou, and Longmenkou hydrological stations (Figure 1). The data on water quality (TN and TP) were derived from the weekly measurement data of raw water samples taken from three river sites and three reservoir sites from March 2019 to October 2021.

The sampling method, determination method, and operation steps of water quality samples were carried out according to the guideline of *Environmental Quality Standards for Surface Water* (GB3838-2002), which is the basic environmental quality standard for surface water issued by the Ministry of Ecological Environment of the People’s Republic of China. In this standard, surface water with Grade III (TN 1 mg/L and TP 0.2 mg/L) can be used as a drinking water source, whereas reservoir or lake water has a lower concentration than the threshold of Grade III, particularly TP (0.05 mg/L).

The measurement method of TN was to convert the nitrogen of the nitrogen-containing compound in the sample into nitrate at 120~124 °C with alkaline potassium persulfate solution and determine it by ultraviolet spectrophotometry. TP was measured by digesting the sample with potassium persulfate under neutral conditions to oxidize all the phosphorus contained in orthophosphate. In an acidic medium, orthophosphate reacted with ammonium molybdate to form phosphomolybdic heteropoly acid in the presence of antimony salt, which was immediately reduced by ascorbic acid to form a blue complex and measured with a spectrophotometer.

For further analysis, rainfall and flow data were summarized in the form of monthly totals, while TN and TP data were summarized in the form of monthly average concentrations. To analyze the various characteristics of water quantity and quality in the Yuqiao Reservoir Watershed in the high flow season, the river and reservoir data were selected from May to October for analysis every year.

The monthly loading calculation formula was as follows:

$$\mathrm{Monthly}\;\mathrm{loading}=k\;\times \overset{D}{\underset{i=1}{\sum }}\frac{\left(C_{i}\;\times \;Q_{i}\;\times \;24\;\times \;60\;\times \;60\right)}{1000}$$

where C_i_ and Q_i_ were the daily mean values of TN or TP (mg/L) and discharge (m^3^/s). Constant *k* is 10^−9^ to convert units from mg to ton (t). D was the total days of each month.

## 3. Results

### 3.1. Rainfall and Hydrology Characteristics

The Sha River has the largest natural flow among the three rivers from May to October, while the flow of the Lin River was the lowest and experienced a long dry time. The water flow of the Li River is controlled by the upstream water transfer. Therefore, Sha River was taken as an example to analyze the changes in runoff in the watershed. The monthly rainfall and flow fluctuation demonstrated that rainfall was a major drive factor in runoff in the Yuqiao Reservoir Watershed.

**Figure 3:**
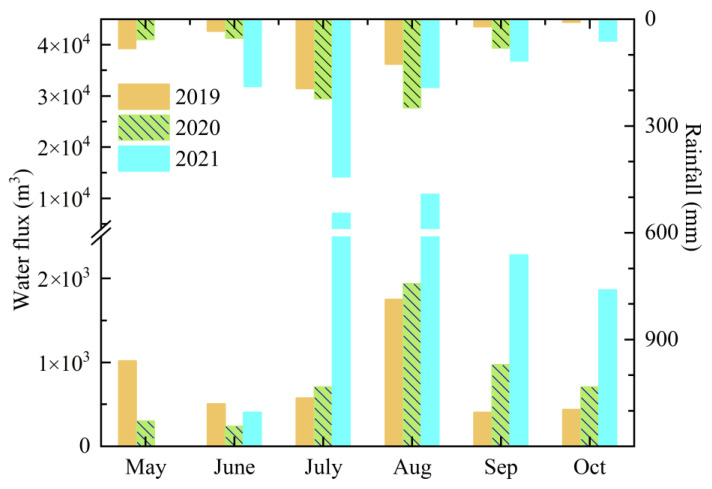
Monthly rainfall and flow of Sha River in high flow season from 2019 to 2021.

Figure 3 showed the rainfall and flow of the Sha River from 2019 to 2021. The rainfall of Sha River Catchment in the high flow season from 2019 to 2021 was 462.6 mm, 661.3 mm, and 999.2 mm, respectively. The produced runoffs of Sha, Li, and Lin Rivers in the same period were 46.8 million m³, 48.5 million m³, and 223.4 million m³ based on the average daily discharge, respectively. In the three years, the high flow season of river flow matched the rainy season and showed a slight delay. Comparing the variation characteristics of monthly rainfall and flow over three years, the rainfall in a normal year was obviously more than that in a dry year (more than 1.4 folds), but the increase of runoff is relatively limited, while both rainfall and runoff in the wet year were obviously increased (nearly two and five times, respectively).

### 3.2. Variation Characteristics of TN and TP Concentrations in Rivers

#### 3.2.1. Variation Characteristics of TN and TP Concentrations in Natural River

**Figure 4:**
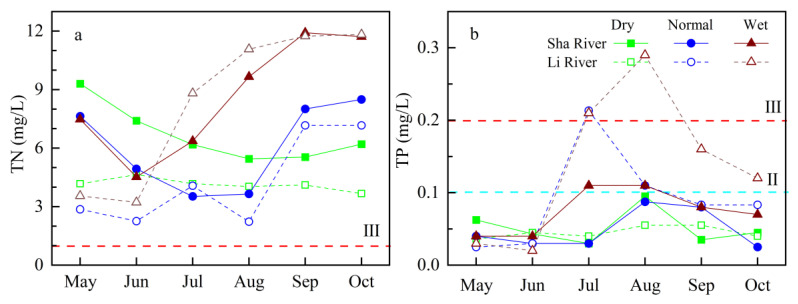
Monthly average TN (**a**) and TP (**b**) concentration changes of Sha River and Li River in high flow seasons from 2019 to 2021.The red and cyan dash line represented the Grade II (TP 0.1 mg/L) and III for surface water basing on the GB3838-2002.

The average concentration of TN in the high flow season of Sha River from 2019 to 2021 was 6.7 mg/L, 6.0 mg/L, and 8.6 mg/L, respectively, corresponding to the TN fluxes 311.7 t, 267.4 t, and 1995.2 t. It can be seen from the data presented in Figure 4a that the TN concentration in the high flow season from 2019 to 2021 showed a decreasing trend first (May to August during dry and normal years, and May to June during the wet year) and then an increasing trend, which is opposite to the change in rainfall and runoff.

During the high flow season of 2019 to 2021, the average concentrations of TP in Sha River were 0.052 mg/L, 0.049 mg/L and 0.075 mg/L, corresponding to TP fluxes of 3.0 t, 3.1 t, and 22.9 t, respectively. TP concentration shows a trend that is rising first and then declining by the end of the wet seasons from 2019 to 2021 (Figure 4b), and this change is very similar to the change in rainfall or flow in the same period. In addition, it can be seen from the total TP in the three years that the TP values in the dry and the normal years are approximately equal, while the TP in the wet year is much greater than that in the previous two years.

#### 3.2.2. Variation Characteristics of TN and TP Concentrations in Water Diversion River

The Li River is an important water diversion channel of “the Water Diversion Project from the Luan River to Tianjin City”. Generally, the natural flow in a wet year is higher than in the dry year and the normal year in the non-diversion period in the high flow season. Figure 4 shows that the concentration patterns of TN and TP in the Li River during the high flow season were different in different years. In the dry year, the concentration range of TN and TP is very small and remains stable. In the normal year, during early May (the water diversion period), the concentration of TN and TP remained at a low level (TN < 0.3 mg/L, TP ≤ 0.030 mg/L), while after the end of the water diversion period in early July, the concentrations of TN and TP increased to a similar concentration to the Sha River in the same period.

### 3.3. TN and TP Characteristics of Yuqiao Reservoir

**Figure 5:**
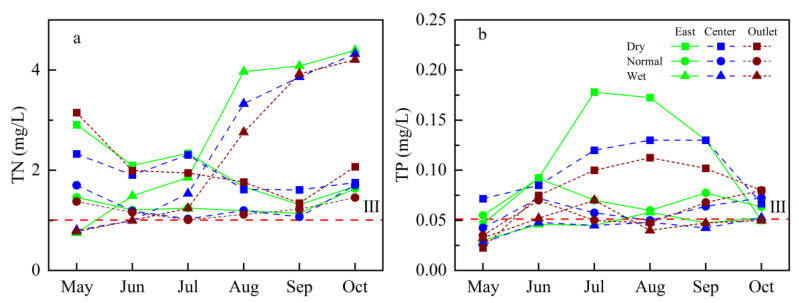
Monthly average TN (**a**) and TP (**b**) concentration changes in the Yuqiao Reservoir in high flow seasons from 2019 to 2021. The red dashed line represents the Grade III for lake or reservoir based on the GB3838-2002.

It can be seen from Figure 5 that the monthly average TN concentrations in three sites of the Yuqiao Reservoir have a similar change trend during the high flow season, but the changing trend of average TN concentration in the wet season in the same region is different in the wet season of different precipitation years. The monthly average TN concentration in the reservoir decreased with time in the high flow season of the dry year, and the average concentration decreased from 2.0 mg/L in June to 1.4 mg/L in September. The fluctuation range of monthly average TN concentration in the high flow season of normal year is limited, and TN concentration in the reservoir is relatively stable. In the high flow season of the wet year, the monthly average TN concentration shows an upward trend. Compared with the range of monthly average TN concentration at different sites of the Yuqiao Reservoir in the same year, it can be found that the spatial variation of monthly average TN concentration in the reservoir is small. Except for the slightly higher monthly average TN concentration in the wet season in the Re-O in 2019, the major spatial characteristics of average TN concentration in the wet season showed a decrease following the flow direction (Re-E > Re-C > Re-O). Comparing the characteristics of TN concentration in the wet season in three years, it can be found that the average TN concentration in the wet season has obvious temporal characteristics of wet year > dry year > normal year, which indicated that overly high or low water flow affects the TN concentration in the reservoir.

The monthly variation characteristics of the average TP concentration in the three sites in Yuqiao Reservoir are similar, but the interannual variation of average TP concentration in the same region during the high flow season is quite different. Monthly average TP concentration changes in the high flow season of the dry year show a trend of rising first and then declining, the concentration shows a large fluctuation, and the concentration increased nearly three times when it was the highest. In the high flow season of the normal year, the monthly average TP concentration changes with a trend of first rising, then falling, and then rising again, whereas the concentration showed a smaller fluctuation. The monthly average TP concentration in the high flow season of the wet year is stable, and only the TP concentration at the Re-O increased slightly in July. In the high flow season of the wet year, the TP concentration has the same spatial variation characteristics as TN, except that the Re-O is slightly higher than the Re-C in 2021. The average TP concentration in the high flow season in the reservoir decreases continuously from the dry year to wet year. This interannual average TP concentration in the high flow season varied, which is inconsistent with the external input and may be caused by the dilution of TP by rainfall in the high flow season rather than the increase of input.

### 3.4. Correlation Analysis

**Figure 6:**
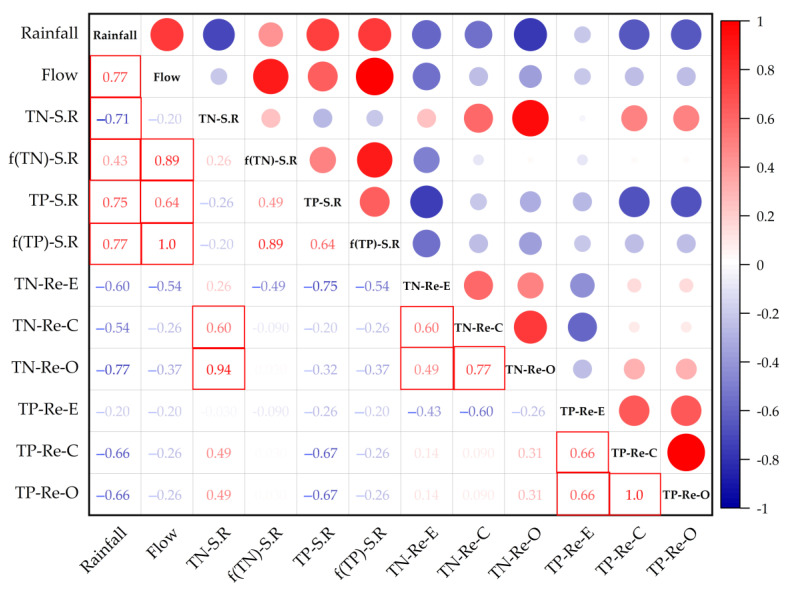
Correlation analysis of variables in the Sha River (S.R) and Yuqiao Reservoir (Re) in the high flow season of 2020. TN and TP represent concentrations of TN and TP; f (TN) and f (TP) represent the flux of TN and TP. A significant correlation is marked by the red boxes.

As similar correlation results in these three years, this study takes the normal year (2020) of the undisturbed watershed (Sha River) and the Yuqiao Reservoir as an example to analyze the correlation between various variables in the watershed in Figure 6. From the correlation between the variables in the watershed in 2020, there is a strong correlation between rainfall and Sha River flow, and Sha River flow can respond well to the changes in rainfall. There is a strong negative correlation between TN concentration and rainfall in the Sha River, whereas a strong positive correlation between TN flux and flow in the Sha River was observed, which indicated that rainfall has a dilution effect on TN in the river and increases the TN load in the Sha River. Rainfall and flow have an obvious positive correlation with the TP concentration and flux of the Sha River, which showed that the increase in rainfall and flow can increase the TP load of the Sha River, indicating P transportation is mainly controlled by the rainfall event flow. In addition, it can be found from Figure 6 that the TN concentration in the river was positively correlated with the TN concentration in the reservoir, while the rainfall was negatively correlated with the TN concentration in the reservoir. Therefore, river input can significantly increase the concentration of TN in the reservoir, and rainfall has a certain dilution effect on the total nitrogen in the reservoir. There is no positive correlation between river TP and reservoir TP, indicating that there are larger sources of TP in the reservoir (such as water diversion and sediment release) than in the inflow rivers. In addition, TN and TP in different areas of the reservoir showed a positive correlation.

## 4. Discussion

### 4.1. Influence Factors of River Water Quality

According to the relationship between rainfall and the flow of the Sha River from 2019 to 2021, rainfall was the main source of natural river water in the Yuqiao Reservoir Watershed. Additionally, rainfall was also the main driving force of non-point source pollution, which can be observed from increased runoff and the obvious increase in TP concentration (R^2^ = 0.80, *p* < 0.01) in the Sha River [30]. Regarding TN concentration, increased runoff in the high flow season mainly diluted the concentration, but the characteristics of TN in river flow varied from a dry to a wet year. For example, the turning point time of TN concentration from decline to rise was gradually ahead. In addition, the time when TN concentration was at the lowest value gradually shortened, while the change range of TN concentration gradually intensified. Although the water source in the normal year was higher than that in a dry year, the TN flux in the dry year was higher than that in the normal year due to the high TN concentration in the dry year.

Previous research in 2008 pointed out that the TN concentration in groundwater in farmland is the highest due to fertilization, while woodland can effectively reduce the TN concentration in groundwater [23]. Therefore, the farmland along the river will significantly impact the river water quality. Urban land is the main factor affecting TN and TP concentrations, particularly point source contribution. Urban construction land will not only produce a large amount of sewage but also, with the increase of the impermeable layer in urban construction, more sewage will flow directly into the river, causing serious pollution [46]. In the present study area, urban land accounts for 25%, which indicates that point sources’ contribution cannot be ignored. In addition, for the garbage produced in rural areas and fecal sewage produced by aquaculture in the watershed, the rainfall washes the surface and generates runoff, which carries these pollutants into the river, causing the deterioration of water quality [47]. Sinha and Michalak [48] analyzed all eight digital hydraulic unit watersheds in the United States based on TN input, rainfall, and land-use models in 2016 and showed that, with the increase in rainfall, the interannual load of TN also increases gradually.

The rivers in Yuqiao Reservoir Watershed showed similar characteristics. With the surge of rainfall in the wet year, the TN load in the river also increases by seven times. Although concentrated rainfall in the high flow season diluted TN concentration for a certain period, the TN load showed an obvious increase. Zhou et al. [49] showed that TP concentration and runoff usually increase with the prolonged rainfall time variation level at all watershed scales. In addition, the larger the runoff, the stronger the migration ability of TP from land to water. The increase in rainfall and flow can also enhance the erosivity and washing effect to mobilize more adsorbed P in the watershed [49]. This also showed that, compared with the increase in rainfall, the increase of TP flux in Sha River is more prominent. For the water diversion river (Li River), the concentrations of TN and TP after the water diversion period in early July were mainly regulated by rainfall. The diversion period in the wet year is mainly concentrated in May and June, and the concentrations of TN and TP also remain at a low level at this stage in May and June. When the diversion period is completed, the concentrations of TN and TP also have a similar change trend to that of the Sha River in the same period.

### 4.2. Influencing Factors of Reservoir Water Quality

Previous studies showed that the diversion, tributary, reservoir perimeter catchment, and reservoir sediment release can affect the water quality of reservoir [38,40,42]. The diversion, tributaries, and the reservoir’s surrounding channel catchments are all external input water bodies that affect the water quality of the reservoir, and rainfall is the main driving force for the rise of TN and TP concentrations in this external input. In addition, rainfall can also contribute pollutants from the atmosphere to the aquatic system [27,50]. This needs attention as a high TN concentration (8.6 mg/L) has been observed in the study area. Therefore, it can be considered that when the land use in the Yuqiao Reservoir Watershed is stable, rainfall is the major reason for the nitrogen and phosphorus output intensity fluctuation in the watershed. Therefore, to prevent the deterioration of reservoir water quality and reduce land-based input, it is necessary to control the source of N and P. Land of different utilization types in the watershed is the main source of non-point source pollution in the Yuqiao Reservoir.

Generally, forest land in its natural state can intercept nutrients, prevent soil erosion, and purify water quality, while agricultural land very easily causes nutrient loss [51]. However, the agricultural land in the Yuqiao Reservoir Watershed accounts for 48% (including 23% of cultivated land and 25% of orchards), which is the largest land use type in the watershed. In addition, when the flow in the wet season is much higher than that in previous years, the nitrogen concentration does not show an obvious dilution effect but is higher, which indicates that there is a large supply of nitrogen source in the watershed. The agricultural land in the basin accounts for nearly 50%, so the contribution of non-point sources cannot be ignored. Therefore, optimizing the application method of agricultural fertilizer, improving sewage treatment capacity, strictly controlling sewage discharge, and improving the level of returning farmland to forests along the river and reservoir are the fundamental measures to improve the water quality of the reservoir [34].

In addition, the release of sediment in the reservoir is mainly related to the concentration of pollutants in sediment and water, their physical and chemical properties, and biological action intensity. Removing sediment at the bottom of the reservoir, blocking the input of exogenous sediment, and regulating the ecosystem structure of the reservoir are important methods to reduce the impact of sediment release on water quality [52,53,54].

As can be seen from the above results, it is concluded that the concentration of TN and TP in the Yuqiao Reservoir has the characteristics of Re-E > Re-C > Re-O. The water level in the Re-E is low and the vegetation grows vigorously [41]. Therefore, when the water flows through the Re-E, aquatic plants may consume part of N and P, thus reducing the concentration of N and P. In addition, Liu et al. [29] showed that for the migration of pollutants in Yuqiao Reservoir, the main flow at the entrance of the reservoir is advection, and when pollutants migrate to the core of the reservoir, diffusion and biochemical processes gradually dominate the process. The transformation of this migration mode leads to the concentration gradient of TN and TP in the reservoir from east to west. In addition, the self-cleaning capacity, the absorption of aquatic plants after the extension of retention time in the reservoir [55], biogeochemical processes, and the removal of sedimentation all regulate the TN and TP concentrations in the reservoir and determine the characteristics of Re-E > Re-C > Re-O [11].

### 4.3. Potential Effect of Climate Change on Water Quality

The study area is in the temperate continental monsoon climate area, which has the characteristics of four distinct seasons and the same period of rain and heat. Climate change will have an impact on rainfall, and rainfall is an important medium to bring pollutants into the water in the watershed [30]. The increase in rainfall directly leads to the increase in the total amount of pollutants containing N and P in the water body, which will further aggravate the degree of eutrophication of the water body [56]. These higher nutrient concentrations will inevitably lead to the deterioration of the water quality of the drinking water source, which should attract more attention. Xiao et al. [57] predicted the rainfall in North China in the future, indicating that the rainfall will increase by nearly 10% by 2040, while the rainfall increase will be as high as 20% by 2080. Therefore, it can be considered that the rainfall in the study area will continue to increase for a long time in the future. Sinha and Michalak [48] simulated the rainfall and TN load changes in the Atchafalaya River Basin in Mississippi through a model. The results showed that only rainfall in the future would increase the TN load in the watershed by 18%, and to offset this increase, the region needs to reduce N input further. Since then, Sinha and others speculated that most parts of East and South Asia, including China and India, will show a similar situation to the Atchafalaya River Basin in the upper Mississippi River in the mainland of the United States in the future [58]. Therefore, to reduce the load of N and P in the watershed and improve water quality, it is very important to reduce the human input of N and P in the watershed.

The increase in rainfall has a certain universality to increasing the TN and TP load of the watershed. Zheng et al. [59] studied the temporal and spatial variation characteristics of TN and TP fluxes in the Yellow River Basin from 2006 to 2017. It is shown that in addition to point source contributions, rainfall is the key driving factor for the interannual variation of TN and TP concentrations in most areas of the Yellow River Basin [59]. Zhou et al. [56] studied the seasonal variation characteristics of water quality of the Jinpen Reservoir in Northwest China and found that the Jinpen Reservoir is very sensitive to storm runoff. Under normal runoff conditions, the inlet water quality is better than that of the main reservoir. However, under storm runoff conditions, the concentrations of TN and TP are higher than those under normal runoff conditions [56]. Li et al. [60] studied the characteristics of water quality changes in the Le’an River Watershed and found that precipitation variability is the leading factor affecting the loss of non-point source nutrients in the Le’an River Watershed, and the weight of climate change caused by precipitation reduction is much greater than land use. Fan and Shibata [61] found that under the climate change scenario, the sediment and nutrient loads will increase in the snowmelt and rainy season. Due to the annual warming in winter, the sediment and nutrient loads will also increase with the increase in winter rainfall [61]. Zhou et al. [49] show that under a given return period and watershed scale, the value of runoff quality and quantity increases with the level of rainfall time variability. At the same time, the response of runoff quality to the spatio-temporal change of rainfall is similar to runoff, and runoff quality is more sensitive than runoff volume. Therefore, if the runoff is generated by rainfall surges, the increase of TN and TP fluxes will be more sensitive to climate change [49]. Overall, it can be concluded that the increase in rainfall will cause more serious water quality problems, which are reflected in many river basins around the world. Therefore, it is urgent to control river basin pollution and improve the water quality of water sources.

To improve the water quality of the watershed, it will be necessary to control pollution emissions and improve the management level of human activities in the watershed. It can be also considered to be important to improve the water quality of lakes and reservoirs by controlling fertilization time, optimizing agricultural fertilization methods, formulating a strict sewage discharge monitoring system, and improving sewage discharge standards. At the same time, improving residents’ awareness of environmental protection and enhancing the sense of responsibility of the whole population to protect the environment is also of great significance to protect water sources.

## 5. Conclusions

This paper studied the changes of TN and TP in the rivers and reservoirs under different rainfall levels during the wet season 2019–2021 in the Yuqiao Reservoir Watershed and analyzed the main factors affecting the water quality in the basin. The results show that rainfall is the main control factor for the inflow of natural rivers into the Yuqiao Reservoir Watershed. The concentrations of TN and TP in natural rivers are regulated by rainfall. The analysis of the influencing factors affecting the water quality of Yuqiao Reservoir Watershed indicated that the non-point source pollution in the basin is the main factor causing the deterioration of river water quality, and the external input with the inflow river into the reservoir is the main source of N and P pollutants in the reservoir. At the same time, the agricultural land in the watershed accounts for nearly 50%, and its impact caused by non-point source pollution cannot be ignored. In addition, the transformation of the migration mode of pollutants in the reservoir and the self-cleaning ability of the reservoir, the absorption of aquatic plants, and biogeochemical processes in the reservoir after the extension of retention time in the reservoir are the main factors causing the spatial differences of TN and TP concentrations in the reservoir. With the increase in rainfall and temperature in North China in the future, N and P loads in the basin will be increased even more so than the present, elevating the pollution load and risk of eutrophication of the aquatic ecosystem. In summary, it is particularly important to control non-point source pollution in the basin, increase forest land coverage in the basin, and reduce the concentration of TN and TP in the reservoir to protect the water quality and safety of drinking water sources.

## Data Availability

The data presented in this study are available upon request from authors.
